# Go/No-Go Ratios Modulate Inhibition-Related Brain Activity: An Event-Related Potential Study

**DOI:** 10.3390/brainsci14050414

**Published:** 2024-04-24

**Authors:** Nan Zhang, Weichao An, Yinghua Yu, Jinglong Wu, Jiajia Yang

**Affiliations:** Graduate of Interdisciplinary Science and Engineering in Health Systems, Okayama University, 3-1-1 Tsushima-Naka, Kita-ku, Okayama 700-8530, Japan; pqqi4ntu@s.okayama-u.ac.jp (N.Z.); pquy1ha9@s.okayama-u.ac.jp (W.A.); yinghua.yyh@gmail.com (Y.Y.); wu@mech.okayama-u.ac.jp (J.W.)

**Keywords:** response inhibition, ratio, go/no-go task, ERP, NoGo-P3 component

## Abstract

(1) Background: Response inhibition refers to the conscious ability to suppress behavioral responses, which is crucial for effective cognitive control. Currently, research on response inhibition remains controversial, and the neurobiological mechanisms associated with response inhibition are still being explored. The Go/No-Go task is a widely used paradigm that can be used to effectively assess response inhibition capability. While many studies have utilized equal numbers of Go and No-Go trials, how different ratios affect response inhibition remains unknown; (2) Methods: This study investigated the impact of different ratios of Go and No-Go conditions on response inhibition using the Go/No-Go task combined with event-related potential (ERP) techniques; (3) Results: The results showed that as the proportion of Go trials decreased, behavioral performance in Go trials significantly improved in terms of response time, while error rates in No-Go trials gradually decreased. Additionally, the NoGo-P3 component at the central average electrodes (Cz, C1, C2, FCz, FC1, FC2, PCz, PC1, and PC2) exhibited reduced amplitude and latency; (4) Conclusions: These findings indicate that different ratios in Go/No-Go tasks influence response inhibition, with the brain adjusting processing capabilities and rates for response inhibition. This effect may be related to the brain’s predictive mechanism model.

## 1. Introduction

The response inhibition refers to an individual’s ability to suppress or delay their response when presented with stimuli. This ability is important in daily life and has high research value in neuroscience [1,2,3]. The neural mechanisms underlying response inhibition can be explored through various experimental paradigms, and the Go/No-Go task is particularly accurate for assessing response inhibition [4,5]. This task typically involves two types of stimuli: Go stimuli, which require a response, and No-Go stimuli, which require response inhibition [6]. Previous studies have focused on response inhibition based on an equal number or specific proportion, such as 7:3, of Go and No-Go conditions. However, the way in which the ratio of Go and No-Go conditions affects the mechanism underlying response inhibition remains unknown.

Reaction times serves as a genuine measure to assess the underlying psychological mechanisms relevant to a psychological experiment. In Go/No-Go experiments, the reaction times to Go stimuli serves as an indicator of the involvement of inhibition processes, exploring the efficiency of inhibition. The slower the reaction times to Go stimuli, the higher the probability of successful inhibition trials, while faster reaction times increase the likelihood of inhibition trial failures [7]. Kok et al.’s study suggests that assumptions regarding the timing and nature of inhibition processes are primarily validated temporally, proving to be reasonable [8]. Several other studies also indicate that models based on the reaction time in Go/No-Go tasks contribute to the interpretability and effectiveness of measuring inhibition mechanisms [9,10,11]. At present, the interpretation of how reaction times to Go stimuli in Go/No-Go tasks under different ratio conditions elucidate inhibition mechanisms is still under exploration.

Event-related potentials (ERPs) allow us to understand how the brain processes different types of stimuli and provide information about individual brain activity during tasks. Currently, there is controversy surrounding ERP studies of response inhibition, and the brain neural mechanisms associated with response inhibition have been investigated. It is generally believed that areas such as the frontal cortex play key roles in the process of response inhibition [12,13]. The positive components around 300 ms (P3) are commonly seen as markers of how the brain evaluates and processes stimuli. Variations in the P3 component indicate the degree to which the brain processes different types of stimuli and allocates cognitive resources relevant to the task. Frontal No-Go-related P3 are typical components that have been widely studied in previous response inhibition studies, although some early studies did not specifically emphasize the relationship between the P3 component and response inhibition. However, in recent years, many studies have indicated that the central P3 components related to No-Go conditions are associated with the process of response inhibition [14,15,16,17]. Albert et al.’s ERP study in particular explored a modified Go/No-Go task composed of stimuli of three different frequency types. The results revealed a greater amplitude of the central P3 in No-Go trials under infrequent conditions compared to Go trials under the same conditions [18]. However, this experiment did not focus on the influence of different Go and No-Go ratio conditions on response inhibition. The different proportions of Go and No-Go stimuli are closely related to the brain’s motor planning and execution, which are crucial for regulating the ability and speed of inhibitory response actions. These processes involve several functional brain areas, such as the primary motor cortex (M1), the pre-motor cortex (PMC), and the supplementary motor area (SMA), which are concentrated in the central region of the brain. The use of ERP technology allows for the capture of relevant brain activity and the differences in brain activity brought by different proportion conditions. Therefore, the present study focused on analyzing the P3 components in central regions and investigating the effects of different ratios of Go and No-Go stimuli on these ERP components.

In the Go/No-Go task paradigm, the ratio of Go and No-Go distributions influences participants’ predictions of stimuli and their ability to inhibit responses, thereby affecting cognitive control and executive function performance [19,20]. For example, when the paradigm includes a higher ratio of Go stimuli, participants are more likely to expect the next stimulus to be a Go stimulus during cognitive processing, making it easier to respond accordingly. Conversely, when the ratio of No-Go stimuli is greater, participants may more frequently anticipate the next stimulus to be a No-Go stimulus, making it easier to inhibit responses [21]. According to the Bayesian brain theory, the brain forms expectations of stimuli and adjusts responses accordingly through stimulus recognition and learning from prior experiences [22]. The human prediction mechanism is based on constantly updating previous knowledge according to new experiences. When external stimuli match expectations, the predictive mechanism strengthens the relevant responses, thereby promoting effective behavioral control. This mechanism plays a crucial role in various cognitive processes, including response inhibition [23,24,25]. In Go/No-Go tasks, the ratio of Go and No-Go distributions is considered a prior probability, representing the initial estimate of the occurrence of different types of stimuli. Therefore, exploring the ratio or probability distribution of Go and No-Go conditions may be valuable in studying the impact of prediction on response inhibition.

Overall, we attempted to modulate the difficulty of the modified Go/No-Go task by introducing directional cues. The high temporal resolution of ERP components enabled us to examine millisecond-level dynamic neural activity. In the experiments, we considered the following four ratio conditions: 100%:0%, 75%:25%, 50%:50%, and 25%:75% proportions of Go and No-Go stimulation. Before starting, participants were informed of the specific distribution in the task description. The experimental task required participants to determine whether the clues and target stimuli were aligned in the same direction. Consistent directions indicated Go trials, while inconsistent directions indicated No-Go trials. We analyzed the reaction time in the Go trials and the error rate in the No-Go trials under different conditions, indicating the influence of prior probability on reaction control. We also analyzed the ERP components, especially the amplitude and latency of the NoGo-P3 component of the central area. The NoGo-P3 component effectively reflects the reaction inhibition process. We assume that as the probability of the Go trials decreases, the reaction inhibition ability weakens, as shown by a decrease in the error rate in the No-Go trials, and the ERP results show a decreased amplitude and a shortened latency for the NoGo-P3 component in the central region.

## 2. Materials and Methods

### 2.1. Participants

Twenty individuals (14 males and 8 females) aged between 20 and 30 years old (with an average age of 24.68 ± 3.15 years, mean ± SD, all right-handed) volunteered for the experiment. None of the participants had a documented history of major medical or neurological issues, such as loss of tactile sensation, epilepsy, severe head injuries, or chronic alcohol dependency. Before taking part in the study, all participants provided written consent. The study protocol was reviewed and approved by the local medical ethics committee at Okayama University in Japan.

### 2.2. Stimuli and Procedures

**Figure 1:**
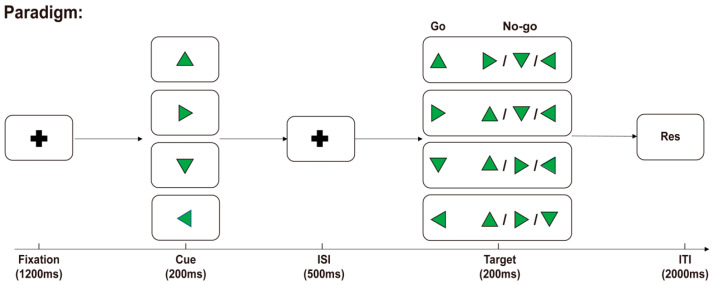
Experimental paradigm. The participants were instructed to promptly assess both stimuli after the presentation of the cue and target. In the “Go” trials, the direction of the stimuli matched, and the joystick on the right-hand side was to be moved in the stimulus direction. In the “No-Go” trials, the directions of the stimuli did not match, and the participants were instructed to refrain from making any movements.

We employed a Go/No-Go task as the experimental paradigm. The experiment was performed in a soundproof chamber utilizing a motion-controlled experimental setup with a 4-way joystick fixed at the right-hand side of the participants. The experimental stimuli were presented on a display screen at a distance of 60 cm from the participants. The experimental paradigm was implemented using MATLAB R2021b, as depicted in Figure 1. Initially, a black central cross was presented for 1200 ms against a gray background (R:127, G:127, B:127), followed by a randomly oriented green equilateral triangle as a visual cue. The time interval between the cue and the target was set at 500 ms. Then, another randomly oriented green equilateral triangle was presented as the target. After the target was presented, participants were required to make an immediate judgment; if the cue and target directions matched, it was deemed a “Go” response; otherwise, it was considered a “No-Go” response. In the “Go” scenario, participants were prompted to swiftly move the joystick toward that direction, while in the “No-Go” scenario, no action was needed. A fixed intertrial interval of 2000 ms followed the conclusion of each target presentation.

The experiment comprised 8 blocks structured as follows: blocks 1 and 2 included 100% Go trials, blocks 3 and 4 included 75% Go trials, blocks 5 and 6 included 50% Go trials, and blocks 7 to 8 included 25% Go trials. The distribution of Go and No-Go trials for each block was as follows: blocks 1 and 2 (Go: 144, No-Go: 0), blocks 3 and 4 (Go: 108, No-Go: 36), blocks 5 and 6 (Go: 72, No-Go: 72), and blocks 7 and 8 (Go: 36, No-Go: 108).

The order of the blocks was randomized. Prior to the start of each block, participants were informed of the ratio of ‘Go’ to ‘No-Go’ stimuli. Following the completion of each block, participants were provided with appropriate rest intervals.

### 2.3. EEG Recording and Preprocessing

The EEG signals were recorded with reference to the left mastoid using a 64-channel amplifier with a sampling frequency of 1000 Hz (Brain Products, Gilching, Germany). And the ground electrode was incorporated into the cap on the medial frontal aspect. Two additional electrodes were placed about 1.5 cm at the left outer canthus and above the right eye to record horizontal and vertical electrooculograms (EOGs), respectively. EEG data were collected with electrode impedances kept below 5 kΩ.

EEG preprocessing was conducted using the EEGLAB (Version 2023.1) and ERPLAB toolboxes (Version 10.01) in MATLAB R2021b. The raw EEG data were bandpass filtered between 0.1 and 30 Hz. Independent component analysis was employed to correct for ocular artifacts. Subsequently, continuous EEG data were down-sampled to 500 Hz and re-referenced to the average of all electrodes. EOGs artifacts were removed. The continuous EEG data were then segmented (−200–800 ms relative to the target). Artifact detection using ERPLAB was performed with all EEG epochs, examining the maximum allowable amplitude difference (threshold: ±100 μV) among all EEG channels within a moving window using the peak-to-peak function. Following artifact rejection, the excluded trials accounted for less than 10% of the total trials, and the trial numbers did not significantly differ across experimental conditions.

### 2.4. Statistical Analysis

Repeated-measures analysis of variance (ANOVA) was used to compare behavioral data and the amplitude and latency of the P3 component at the average of the central electrodes (Cz, C1, C2, FCz, FC1, FC2, PCz, PC1, PC2) using SPSS 26.0. Correct responses in the Go and No-Go trials were of interest. If Mauchly’s test of sphericity was violated, the degrees of freedom were adjusted using Greenhouse–Geisser correction. For behavioral data, we compared the mean reaction times in the Go trials and the error rates in the No-Go trials under different ratio conditions. The time window for the NoGo-P3 component at the average of the central electrodes was set between 300 ms and 400 ms, with Bonferroni corrections applied for multiple comparisons. Statistical significance was accepted at *p* < 0.05. Unless otherwise stated, all results are presented as the mean ± MSE (standard error of the mean).

## 3. Results

### 3.1. Behavioral Performance

**Figure 2:**
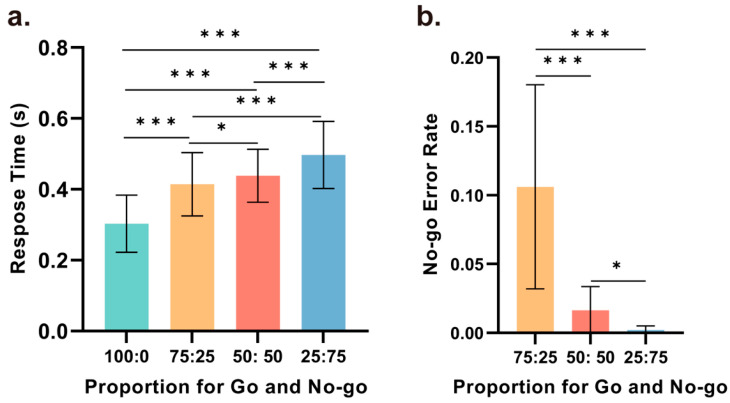
Behavioral performance. (**a**) A comparison of the reaction times for Go trials between any two ratio conditions revealed statistically significant differences. Reaction times were shortest when Go trials comprised 100% of the trials and longest when Go trials comprised 25% of the trials. Statistical significance was observed for all comparisons between any two conditions. (**b**) A comparison of the error rate for No-Go trials between any two ratio conditions revealed statistically significant differences. The error rates were highest with a Go trial ratio of 75%, whereas a Go trial ratio of 25% resulted in the lowest error rates. Statistical significance was observed for all comparisons between any two conditions (* *p* < 0.05, *** *p* < 0.001).

We conducted repeated-measures ANOVA based on the reaction times in all correct Go trials under the four different Go and No-Go ratio conditions (with Go trial ratios of 100%, 75%, 50%, and 25%). The statistical analysis results showed that the main effect of the reaction time in the Go trials across the four conditions was significant (*F* (2.177, 21.775) = 59.723, *p* < 0.001, η_p_^2^ = 0.857), with significant differences observed between any two conditions. As depicted in Figure 2a, the shortest reaction times were observed with a Go trial ratio of 100%, with the reaction time gradually increasing as the ratio decreased. Compared to the 100% Go condition, the reaction times were significantly increased in the 75% (*t*(21) = 5.830, *p* < 0.001, *d* = 1.758), 50% (*t*(21) = 8.850, *p* < 0.001, *d* = 2.668), and 25% conditions (*t*(21) = 11.024, *p* < 0.001, *d* = 3.324). Moreover, the reaction times in the 75% (*t*(21) = 6.286, *p* < 0.001, *d* = 1.895) and 50% (*t*(21) = 4.932, *p* < 0.001, *d* = 1.487) conditions were significantly shorter than that in the 25% condition. Additionally, while the difference in reaction time between the 50% and 75% conditions was not as pronounced, the difference was still statistically significant (*t*(21) = 2.338, *p* = 0.041, *d* = 0.705). 

Furthermore, repeated-measures ANOVA was conducted based on the error rates in the No-Go trials under three different ratio conditions (with Go trial ratios of 75%, 50%, and 25%). The main effect of the error rate in the No-Go trials across the three conditions was significant (*F* (1.028, 10.280) = 23.21, *p* = 0.001, η_p_^2^ = 0.699), with significant differences observed between any two conditions. As illustrated in Figure 2b, the error rates were highest with a Go trial ratio of 75%, and the error rate in this condition was significantly greater than the error rate in the 50% (*t*(21) = 4.928, *p* < 0.001, *d* = 0.1.485). and 25% conditions (*t*(21) = 4.796, *p* < 0.001, *d* = 1.446). Under the 25% condition, the error rate in the No-Go trials was nearly zero, which was significantly lower than that in the 50% condition (*t*(21) = 3.203, *p* = 0.028, *d* = 0.966).

### 3.2. ERP Results

**Figure 3:**
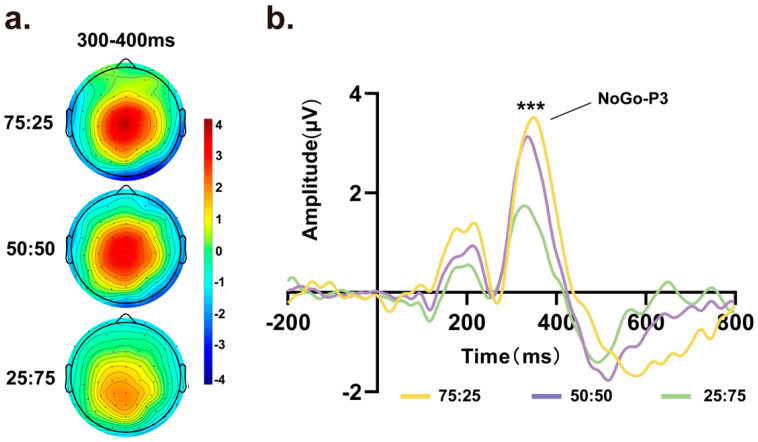
(**a**) Scalp topographic maps and waveform graphs of the average of the central electrodes (Cz, C1, C2, FCz, FC1, FC2, PCz, PC1, PC2) from 300 ms to 400 ms. In the scalp topographic map, variations in the central area can be observed under different Go and No-Go ratio conditions, with lower Go trial proportions associated with lower amplitudes. (**b**) Target-related ERPs of the average central electrodes, with a time window of 0–800 ms. Significant differences in the amplitude of the NoGo-P3 component were observed under different conditions. (*** *p* < 0.001).

For the NoGo-P3 component, as there were no No-Go trials in the 100% condition, we analyzed No-Go trial data in the other three conditions (75%, 50%, and 25% Go trials). As shown in Figure 3a, scalp topographical maps of target stimuli were obtained within an 800 ms time window, revealing prominent signal variations at approximately 300 ms to 400 ms at central locations among the different proportion conditions. As the proportion of Go trials decreased, the scalp voltage in the No-Go trials decreased accordingly. Next, we extracted data from the average of Cz, C1, C2, FCz, FC1, FC2, PCz, PC1 and PC2 to generate waveform plots, as depicted in Figure 3b. Repeated-measures ANOVA was conducted on the amplitude of the NoGo-P3 component, indicating a significant main effect of amplitude among the three proportion conditions (*F* (1.305, 27.406) = 37.113, *p* < 0.001, η_p_^2^ = 0.639). In addition, repeated-measures ANOVA was conducted on the latency, which also showed a significant main effect among different proportions of Go and No-Go trials (*F* (1.345, 28.243) = 7.537, *p* = 0.006, η_p_^2^ = 0.264).

**Figure 4:**
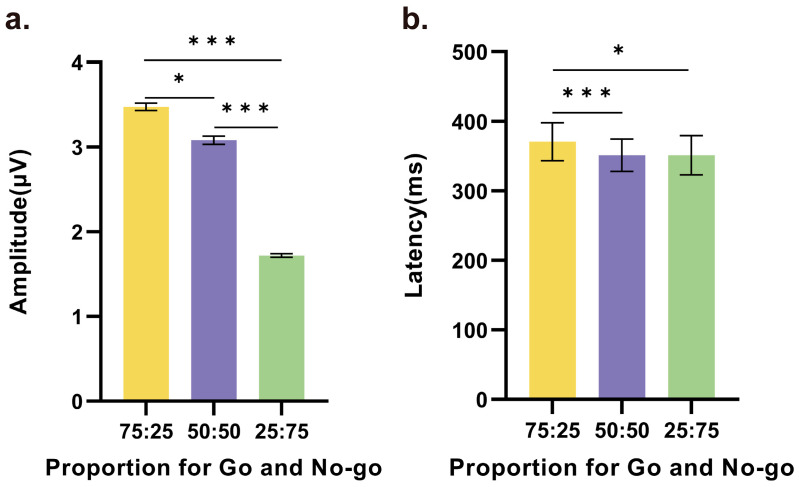
Significant differences in the (**a**) amplitude and (**b**) latency of the NoGo-P3 component based on the average of the central electrodes among the three conditions. Statistically significant differences were observed for nearly all comparisons between any two conditions, except for the latency between 50% and 25% (* *p* < 0.05, *** *p* < 0.001).

Furthermore, pairwise comparisons between any two conditions revealed statistically significant differences in both the amplitude and latency of the NoGo-P3 component. In particular, as shown in Figure 4a, the amplitude in the 75% Go condition was higher than those in the 50% (*t*(21) = 2.787, *p* = 0.011, *d* = 0.594) and 25% conditions (*t*(21) = 6.801, *p* < 0.001, *d* = 1.450), and the second highest amplitude, which was observed in the 50% condition, was significantly higher than the amplitude in the 25% condition (*t*(21) = 2.104, *p* < 0.001, *d* = 2.104). In addition, as shown in Figure 4b, there was no difference in the latency of the NoGo-P3 component between the 50% and 25% conditions. However, in the 75% condition, the average peak occurred later than those in the 50% and 25% conditions, and these times were significantly different (75% vs. 50%: *t*(21) = 4.219, *p* < 0.001, *d* = 0.900, 75% vs. 25%: *t*(21) = 2.812, *p* = 0.010, *d* = 0.599).

## 4. Discussion

In this study, we explored the effects of the ratio of Go and No-Go trials on response inhibition. The results revealed that the reaction times significantly increased as the proportion of Go trials decreased. Furthermore, the error rate in the No-Go trials gradually decreased, approaching zero in the 25% Go trial condition. The ERP results also aligned with our hypothesis; specifically, significant differences were observed in the amplitude and latency of the NoGo-P3 component based on the average of the central electrodes (Cz, C1, C2, FCz, FC1, FC2, PCz, PC1, and PC2) under different conditions. As the proportion of Go trials decreased, the amplitude and latency of the NoGo-P3 component gradually decreased. These findings indicate that the ratio of Go to No-Go trials affects the efficiency and capability of response inhibition.

During Go/No-Go tasks, different ratio conditions served as prior information for participants when predicting their responses. Initially, participants compared the directions of the cue and target during the experiment. Previous studies suggest that during this process, the brain’s working memory mechanism is used to memorize and compare information [26,27]. In the Go condition, participants pushed the joystick toward the response direction, while response inhibition occurred in the No-Go condition. In the 100% Go trial condition, participants needed to differentiate and remember only the response direction, resulting in the fastest decision-making and action execution processes. However, as the proportion of Go trials decreases, more attention is needed to distinguish trial types, leading to slower processing speeds and increased reaction times in Go trials. Concurrently, the brain forms expectations based on the ratio of information about upcoming target stimuli, transitioning from expecting response inhibition in the No-Go condition to expecting a response in the Go condition. Our findings are closely related to Gavazzi et al.’s recent study, which is based on the average reaction times to Go stimuli from 68 Go/No-Go studies and established a model reflecting the demand level of inhibitory control mechanisms. This model utilized the average likelihood estimation (ALE) meta-analysis algorithm and ES-SDM meta-regression to employ the mean and standard deviation of sample reaction times as linear predictor factors in three meta-regression models. The results revealed a negative correlation between average reaction time and activation in the right frontal lobe. These findings suggest that assessing Go reaction times as indicators of involvement in the inhibition process enhances our understanding of the neural correlates of cognitive control for achieving inhibition [7]. The variation in reaction times under Go conditions can effectively explain the differences in the level of response inhibition brought about by expectancy. The error rate in the No-Go trials reflects participants’ lack of control in response inhibition [28,29]. As the proportion of No-Go trials increased, the error rate decreased, indicating enhanced attention and inhibition abilities during information processing. This enhancement is related not only to adjustments after errors occur but also to expectations based on ratio information.

The electrodes in the central region, including those we recorded, such as Cz, C1, C2, FCz, FC1, FC2, PCz, PC1, and PC2, capture the NoGo-P3 component, effectively assessing the impact of different ratio conditions on response inhibition in Go/No-Go tasks. The central region typically encompasses areas such as the parietal lobe, frontal lobe, primary sensory cortex, and motor cortex [14,30,31,32]. Electrodes distributed in these areas can detect potential activities related to various motor control and cognitive functions with high temporal precision, providing valuable insights into the neural activities underlying cognitive processing mechanisms and response inhibition [33,34]. A larger NoGo-P3 amplitude is often interpreted as a stronger response inhibition capability, while a shorter latency may suggest faster response inhibition [35,36]. The topographical maps generated between 300 ms and 400 ms after presenting the target stimulus indicate that the main site for processing information related to NoGo-P3 is near the central region, and the amplitude of this component decreased as the proportion of No-Go trials increased. Moreover, the waveforms show that as the proportion of No-Go trials increased, the amplitude and latency of the NoGo-P3 component significantly decreased at the average electrode in the central region. This suggests that the response inhibition capability was reduced, while the speed of response inhibition was increased. Some studies also provide substantial support for our research. Albert et al. found that the NoGo-P3 component in the central region under infrequent No-Go conditions is effective in measuring brain activity related to response inhibition [18]. Smith et al. discovered that the NoGo-P3 effect is attributed to cognitive or non-motor inhibition [32]. Gajewski et al.’s research linked the NoGo-P3 to inhibiting motor responses [37]. However, to our knowledge, there are few studies using ERP techniques to assess the impact of different ratios of Go and No-Go stimuli on response inhibition. Different stimulus ratios trigger processes related to the brain’s motor planning and prediction, crucial for regulating the ability and speed of inhibitory response actions. These processes involve several functional brain areas, such as the M1, the PMC, and the SMA, concentrated in the central region of the brain. Our results also validate the credibility of our hypothesis. Therefore, the NoGo-P3 component in the central region can be used to effectively assess the impact of trial ratios conditions on response inhibition in Go/No-Go tasks.

In Go/No-Go tasks, the ratio of Go and No-Go trials may lead to differences in the levels of response inhibition, which could be related to the brain’s predictive mechanisms. Prediction and response inhibition are both abilities that involve control, and predictive abilities may affect the effectiveness of response inhibition [21]. The brain forms expectations by recognizing stimuli and learning from prior knowledge and then adjusts its responses accordingly. When external stimuli match expectations, the predictive mechanism strengthens relevant responses, aiding effective behavioral execution [38]. The Bayesian brain model applies this predictive mechanism, suggesting that humans use Bayesian-like reasoning when processing uncertain information, continuously updating information based on prior knowledge and new experiences [22,39]. Therefore, the brain adjusts its responses based on prior information [40,41]. In Go/No-Go tasks, the ratio of Go and No-Go trials serves as prior information, representing initial estimates of the occurrence of different types of stimuli, triggering such predictive mechanism [20]. When the probability of Go stimuli is higher, participants may be more inclined to predict the next stimulus as a Go stimulus, making it easier to respond accordingly. Conversely, when the probability of No-Go stimuli is higher, it is easier to inhibit responses. Therefore, different ratios of Go and No-Go trials can influence participants’ abilities to predict stimuli and inhibit responses, consequently affecting cognitive control and executive function.

## 5. Conclusions

In summary, this study suggests that different ratios of Go and No-Go trials in Go/No-Go tasks modulate response inhibition. The brain adjusts inhibition capability and the processing rate of inhibitory responses based on this ratio information, which serves as prior knowledge. This modulation of response inhibition by the trial ratio can be observed based on changes in the amplitude and latency of the NoGo-P3 component recorded at electrodes in the central region (Cz, C1, C2, FCz, FC1, FC2, PCz, PC1, and PC2) during Go/No-Go tasks. As the proportion of No-Go trials increases, the amplitude and latency of the NoGo-P3 component decrease, indicating reduced response inhibition capability and slower processing of information related to response inhibition. This study expands the application of the Go/No-Go paradigm. Moreover, the study results are crucial for understanding and exploring the neural mechanisms underlying response inhibition and suggest promising directions for future research on modulating response inhibition.

## Data Availability

The data are not publicly available because further analysis for this research is still ongoing, but the data are available on reasonable request from the corresponding author.
